# Martius’ flap for recurrent perineal and rectovaginal fistulae in a patient with Crohn’s disease, endometriosis and a mullerian anomaly

**DOI:** 10.1186/s12893-017-0309-8

**Published:** 2017-11-21

**Authors:** Gaetano Gallo, Alberto Realis Luc, Giuseppe Clerico, Mario Trompetto

**Affiliations:** 1 Department of Colorectal Surgery, Santa Rita Clinic, Vercelli, Italy; 2 Department of Surgical and Medical Sciences, University “Magna Graecia” of Catanzaro, Catanzaro, Italy

**Keywords:** Rectovaginal fistula, Crohn’s disease, Mullerian anomalies, Martius’ flap, Dyspareunia

## Abstract

**Background:**

Rectovaginal fistulas represent 5% of all anorectal fistulae and are a disastrous manifestation of Crohn’s disease that negatively affects patients’ social and sexual quality of life. Treatment remains challenging for colorectal surgeons, and the recurrence rate remains high despite the numerous available options.

**Case presentation:**

We describe a 31-year-old female patient with a Crohn’s disease-related recurrent perineo-vaginal and recto-vaginal fistulae and a concomitant mullerian anomaly. She complained of severe dyspareunia associated with penetration difficulties. The patient’s medical history was also significant for a previous abdominal laparoscopic surgery for endometriosis for the removal of macroscopic nodules and a septate uterus with cervical duplication and a longitudinal vaginal septum. The patient was successfully treated using a Martius’ flap. The postoperative outcome was uneventful, and no recurrence of the fistula occurred at the last follow-up, eight months from the closure of the ileostomy.

**Conclusion:**

Martius’ flap was first described in 1928, and it is considered a good option in cases of rectovaginal fistulas in patients with Crohn’s disease.

The patient should be referred to a colorectal centre with expertise in this disease to increase the surgical success rate.

## Background

Crohn’s disease (CD) is the second most common cause of a rectovaginal fistula (RVF) after obstetrical trauma [1, 2], and it is likely the primary risk factor for fistula recurrence [3].

A RVF represents 9% of all fistulas in CD, and it affects approximately 5 to 10% of female patients [4, 5]. A RVF significantly affects the patient’s psychosocial and sexual quality of life.

Fistulizing CD is a predictor for stoma construction [6], and its clinical manifestations include vaginal passage of air or faecal material, bleeding, abscess formation, perianal pain, dyspareunia, vaginal irritation and recurrent genitourinary infections [7–10].

Patients often require multiple surgical attempts, on average three in our experience, to achieve a satisfactory result, and a step-up approach is the current standard [11, 12].

A flap using the bulbocavernous muscle was first described in 1928 by Dr. Heinrich Martius, a professor of gynaecology in Gottingen, for urethral reconstruction, and this approach must be considered in cases of recurrent or CD-related RVFs [13]. The major drawback of this technique is post-operative dyspareunia, which occurs in 30% of cases. The incidence of labial wound issues is less than 10% [14, 15].

We describe the preoperative and operative details of Martius’ flap in the management of a 31-year-old female with CD-related recurrent perineovaginal and RVF with a concomitant mullerian anomaly.

## Case presentation

**Fig. 1:**
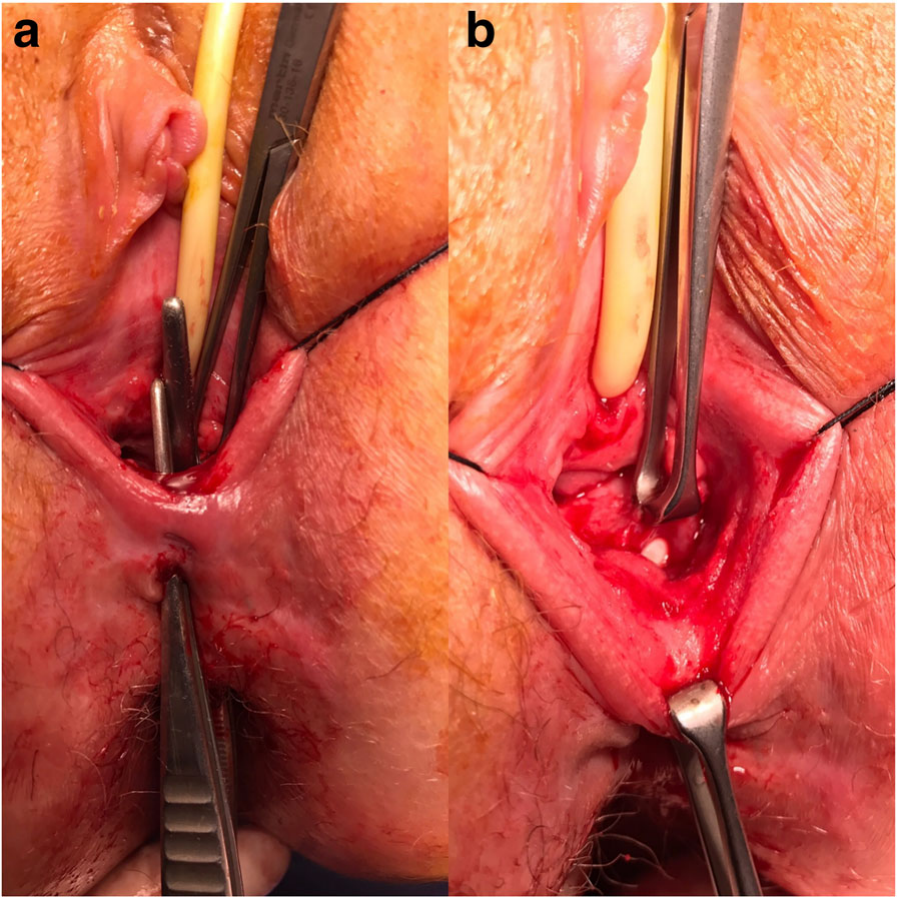
**a** The fistulae are indicated by the two forceps passing through them; **b** The finger demonstrates the large recto-vaginal orifice

**Fig. 2:**
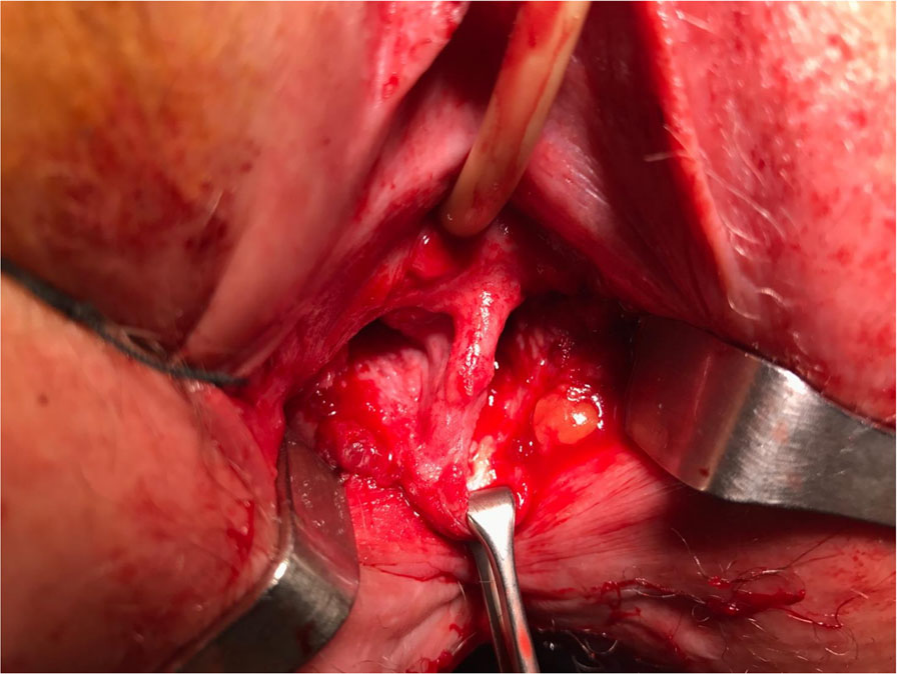
Longitudinal vaginal septum

A 31-year-old female with CD was referred for perineovaginal and RVF (2 cm from the vaginal introitus and 2 cm in diameter) (Fig. 1 a, b). Previous direct repairs of the fistula using vaginal and rectal approaches failed. The patient recently underwent small bowel resection with a loop ileostomy. The medical history of the patient was also significant for a previous abdominal laparoscopic surgery for endometriosis with removal of macroscopic nodules and a septate uterus with cervical duplication and a longitudinal vaginal septum (Fig. 2). She complained of severe dyspareunia associated with penetration difficulties and the well-known disturbances of the RVF.

## Operative details

All patients with RVFs in our department undergo preoperative MRI, pancolonoscopy, and full mechanical bowel preparation and receive antibiotic and antithrombotic prophylaxis and an endoanal ultrasound (EUS) scan to visualize and map the sublinical concomitant sphincter defect.

The quality of the sex life and continence levels of the patient are assessed using the Female Sexual Function Index (FSFI) [16, 17] and the Cleveland Clinic Incontinence Score (CCIS) [18], respectively. We always perform an evaluation under anaesthesia (EUA) to plan a tailored surgery for each specific patient.

## Surgical technique

The Martius’ procedure was performed under spinal anaesthesia with the patient in the lithotomy position. A temporary urinary catheter was preoperatively placed and removed at the end of the procedure.

**Fig. 3:**
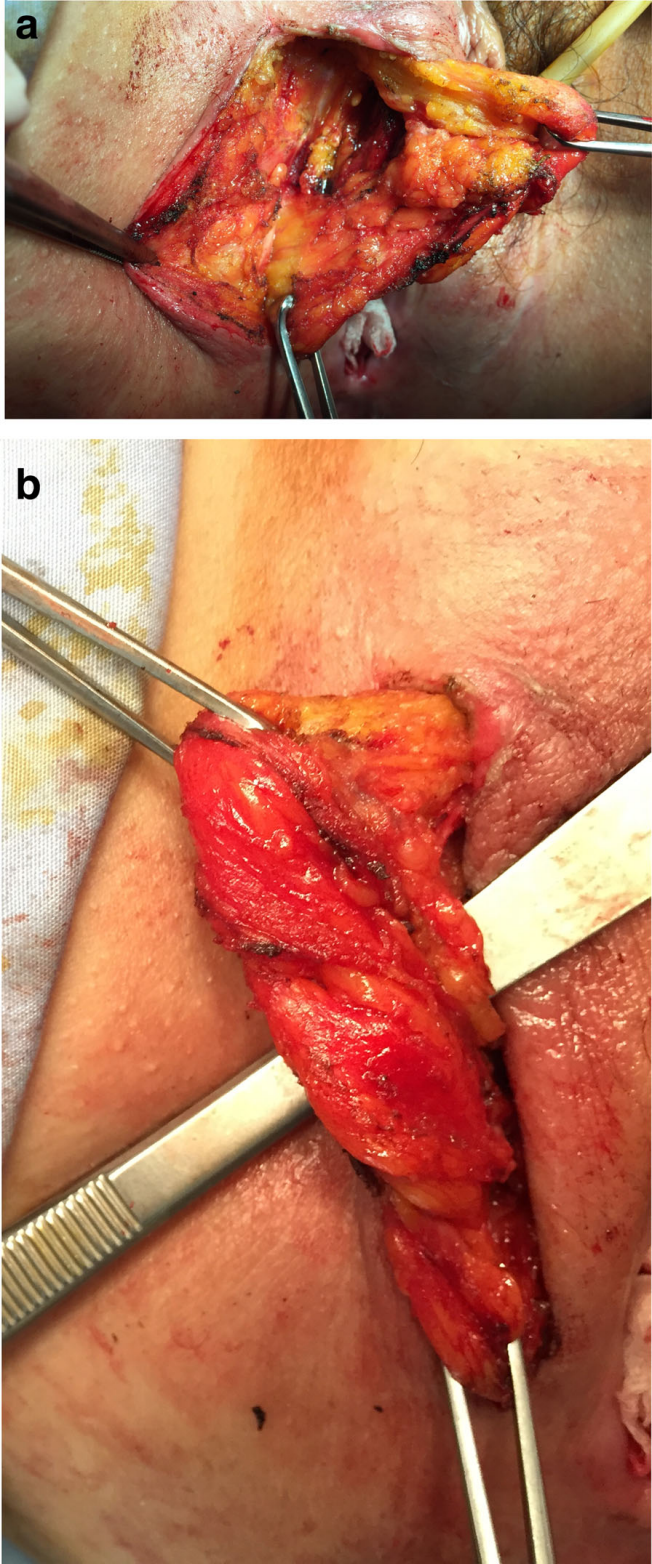
**a**, **b** After a right labial incision, the bulbocavernous muscle and the surrounding fibroadipose tissue were carefully mobilized, avoiding possible damage of the postero-external vascular pedicle

**Fig. 4:**
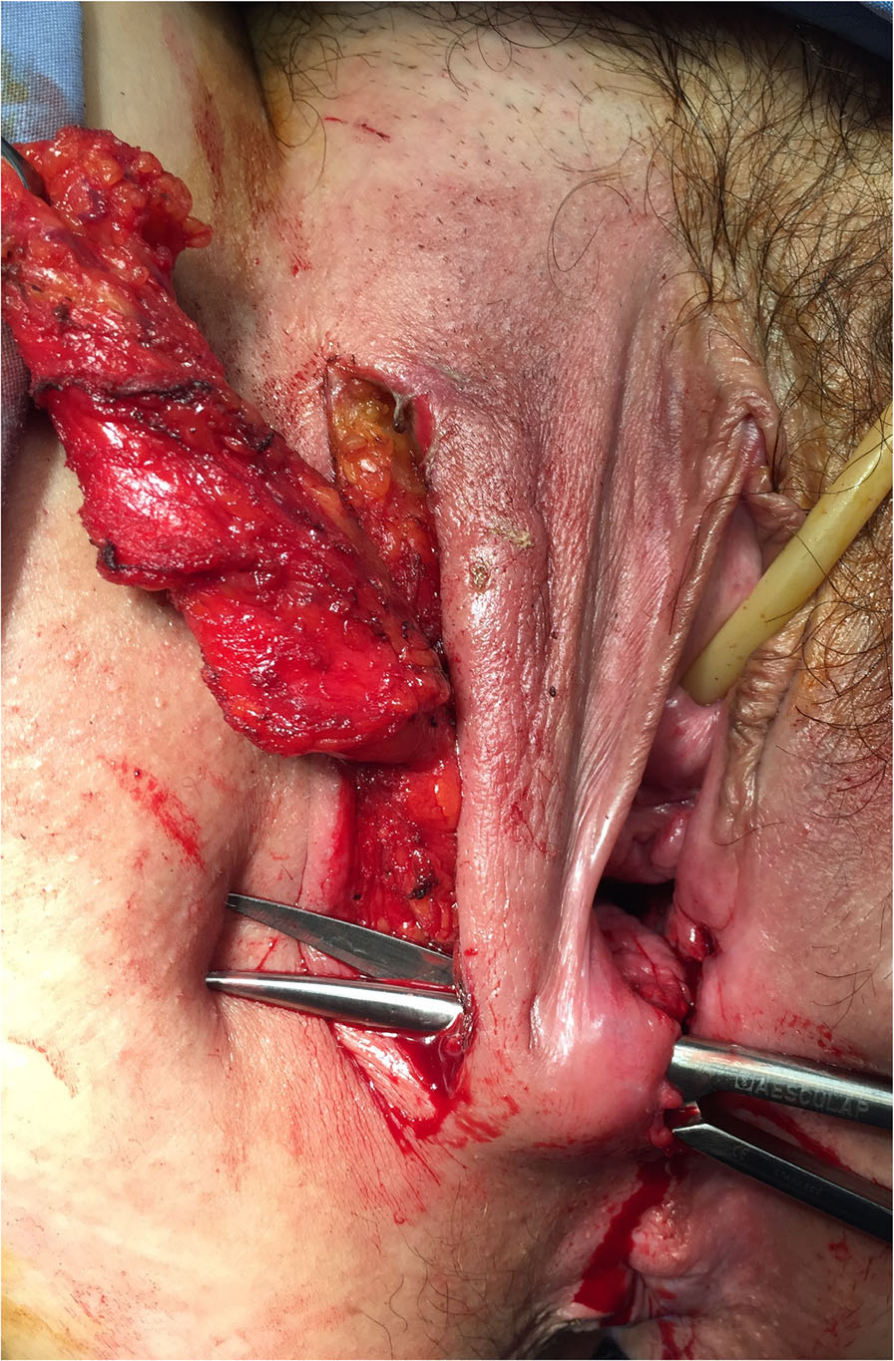
A subcutaneous tunnel connecting the two incisions was created after transecting superior to the bulbocavernous muscle

**Fig. 5:**
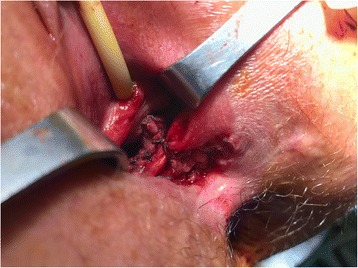
The final set-up with the interrupted absorbable sutures over the vaginal closure after sectioning of the longitudinal vaginal septum. The bulbocavernous muscle is clearly visible after the lay open of the perineovaginal tract

Fistula tracts were identified using a conventional fistula probe. A transverse incision 3-4 cm in length at the vaginal introitus and a careful dissection of the rectovaginal septum were performed, freeing it up to 3-4 cm above the fistula to enable a tension-free repair. The orifice of the fistula was curetted and closed from the vaginal side using interrupted 2-0 absorbable sutures. A vertical left paravaginal incision 7 cm in length enabled individualization of the bulbocavernous muscle, which was carefully isolated to preserve the postero-external vascular pedicle derived from the internal pudendal artery (Fig. 3 a, b). A subcutaneous tunnel (Fig. 4) was created to transpose the pedunculated muscle into the previously prepared rectovaginal plane to fill the dead space and protect the direct repair. The graft was fixed using an interrupted 2-0 absorbable suture. A contemporary lay open of the superficial perineovaginal fistula (Fig. 5) followed by a concomitant partial resection of the longitudinal vaginal septum leaving both cervixes separated in the vaginal fundus was performed.

The vertical paravaginal incision was closed with absorbable suture, and a Penrose drain was left in place. The vaginal opening was left open, and packing gauze was removed on the first post-operative day.

The need to create a temporary diverting stoma to increase the percentage of surgical success rate of treating a RVF remains controversial [19]. Faecal diversion is mandatory in patients with a large, recurrent or CD-related RVF because it improves the chances of satisfactory results as an independent factor for success [20], avoids wound infection and solves the possibility of acute perianal sepsis. A loop ileostomy due to previous small bowel resection was already in place in our case.

The patient began oral nutrition the day after surgery and received intravenous short-term antibiotic therapy with metronidazole and third-generation cephalosporin. She was discharged two days after the procedure.

The post-operative outcome was uneventful, and after a new EUA demonstrating the closure of the RVF, the loop ileostomy was closed three months after the first surgery.

No recurrence of the fistula occurred at the last follow-up eight months from closure of the ileostomy. The significant subjective satisfaction of the patient for sexual function was confirmed as improvement of the FSFI from 4.7 to 22.3, and normal faecal continence (CCIS = 0) was maintained.

## Discussion

RVFs related to CD are challenging for surgeons, and a very high propensity to recur is observed, with published rates ranging from 25% to 80% [7, 21, 22]. An optimal medical or surgical control of local and distant CD acute/subacute manifestations must be achieved. Some studies suggest that a previous seton placement and faecal diversion improves the rate of successful fistula closure via reduction of local inflammation and infection [23, 24].

The interposition of healthy, well-vascularized tissue, such as the bulbocarvernous muscle [25, 26] with its surrounding fibroadipose component that protects the direct repair and separates the rectal and vaginal walls, may be a good option in these particularly difficult cases.

Pitel et al. reported a successful healing rate of 50% for CD-related RVFs in the largest study of Martius’ flap for RVFs [27].

An important trick to improve the success of the procedure is the careful mobilization of the muscle to avoid possible damage of the postero-external vascular pedicle and excessive tension of the graft, which may result in a subsequent migration.

A septate uterus, double cervix, and longitudinal vaginal septum are uncommon, and the true incidence of this triad is unknown [28]. This anomaly represents a failure in the fusion of the distal Mullerian ducts and results in dysmenorrhoea, dyspareunia and infertility, which are the main symptoms in most patients.

Notably, this case of the use of a Martius’ flap significantly decreased the dyspareunia, which is commonly a frequent side effect of the procedure [14, 15].

## Conclusions

RVFs in CD are a frequent and debilitating condition.

No ideal treatment exists for this condition, despite the numerous medical and surgical options proposed for the treatment of RVFs, and the recurrence rate remains high.

An appropriate surgical treatment should be carefully chosen and individualized according to the anatomy and aetiology of the fistula, patient symptoms, comorbidities and previous repairs.

Therefore, patients complaining of a RVF should be referred to specialized centres with a special interest and experience in this disease [11, 29–31].

The use of a Martius’ flap is one approach in cases of patients with a large, recurrent RVF or when there is no possibility of using the periorificial tissue due to local severe inflammation or fibrosis.

The need for faecal diversion, prior or concomitant to the repair, depends on the surgeon’s judgement, but it is mandatory for recurrent and CD-related RVF in our opinion.

## References

[1] Hannaway CD, Hull TL (2008). Current considerations in the management of rectovaginal fistula from Crohn’s disease. Color Dis.

[2] Andreani SM, Dang HH, Grondona P, Khan AZ, Edwards DP (2007). Rectovaginal fistula in Crohn’s disease. Dis Colon rectum.

[3] Pinto RA, Peterson TV, Shawki S, Davila GW, Wexner SD (2010). Are there predictors of outcome following rectovaginal fistula repair?. Dis Colon rectum.

[4] Schwartz DA, Loftus EV, Tremaine WJ (2002). The natural history of fistulizing Crohn’s disease in Olmsted County, Minnesota. Gastroenterology.

[5] Radcliffe AG, Ritchie JK, Hawley PR, Lennard-Jones JE, Northover JM (1988). Anovaginal and rectovaginal fistulas in Crohn’s disease. Dis Colon rectum.

[6] Geltzeiler CB, Hart KD, Lu KC, Deveney KE, Herzig DO, Tsikitis VL (2015). Trend in the surgical Management of Crohn’s disease. J Gastrointestinal Surg.

[7] Valente MA, Hull TL (2014). Contemporary surgical management of rectovaginal fistula in Crohn’s disease. World J Gastrointes Pathophysiol.

[8] Sand BE (2004). Long-term treatment of rectovaginal fistulas in Crohn’s disease: response to infliximab in the ACCENT II study. Clin Gastroenterol Hepatol.

[9] de la Poza G, Lopez-Sanroman A, Taxonera C (2012). Genital fistulas in female Crohn’s disease patients: clinical characteristics and response to therapy. J Crohns Colitis.

[10] Das B, Snyder M (2016). Rectovaginal Fistulae. Clin Colon Rectal Surg.

[11] Pescatori M (2012). Prevention and treatment of complications in proctological surgery.

[12] Zhu YF, Tao GQ, Zhou N, Xiang C (2011). Current treatment of rectovaginal fistula in Crohn’s disease. World J Gastroenterol.

[13] Martius H (1928). Die operative Wiederherstellung der vollkommen fehlenden Harnrohare und des Schliessmuskels derselben. Zentralb Gynakol.

[14] McNevin MS, Lee PY, Bax T (2007). Martius flap: and adjunct for repair of complex, low rectovaginal fistula. Am J Surg.

[15] Kniery KR, Johnson EK, Steele SR (2015). Operative considerations for rectovaginal fistulas. World J Gastrointest Surg.

[16] Rosen R, Brown C, Heiman J (2000). The female sexual function index (FSFI): a multidimensional self-report instrument for the assessment of female sexual function. J Sex Marital Ther.

[17] Wiegel M, Meston C, Rosen R (2005). The female sexual function index (FSFI): cross validation and development of clinical cutoff score. J Sex Marital Ther.

[18] Rochwood TH, Church JM, Fleshman JW (2000). Fecal incontinence quality of life scale: quality of life instrument for patients with fecal incontinence. Dis Colon rectum.

[19] Lambertz A, Lüken B, Ulmer TF (2016). Influence of diversion stoma on surgical outcome and recurrence rates in patients with rectovaginal fistula – a retrospective cohort study. Int J Surg.

[20] Corte H, Maggiori L, Treton X, Lefevre JH, Ferron M, Panis Y (2015). Rectovaginal fistula: what is the optimal strategy? An analysis of 79 patients undergoing 286 procedures. Ann Surg.

[21] El Gazzaz G, Hull TL, Mignanelli E, Hammel J, Gurland B, Zutshi M (2010). Analysis of function and predictors of failure in women undergoing repair of Crohn's related rectovaginal fistula. J Gastrointest Surg.

[22] Narang R, Hull T, Perrins S, Garcia JS, Wexner SD (2016). Should Immunomodulation therapy Alter the surgical Management in Patients with Rectovaginal Fistula and Crohn’s disease?. Dis Colon rectum.

[23] Zmora O, Tulchinsky H, Gur E (2006). Gracilis muscle transposition for fistula between the rectum and urethra or vagina. Dis Colon rectum.

[24] Lowry AC, Thorson AG, Rothenbeger DA, Goldberg SM (1988). Repair of simple rectovaginal fistulas: influence of previous repairs. Dis Colon rectum.

[25] Kniery K, Johnson EK, Steele SR (2015). How I do it: Martius flap for rectovaginal fistulas. J Gastrointest Surg.

[26] Reichert M, Schwandner T, Hecker A (2014). Surgical approach for repair of Rectovaginal fistula by modified Martius flap. Geburtshilfe Frauenheilkd.

[27] Pitel S, Lefevre JH, Parc Y, Chafai N, Shields C, Tiret E (2011). Martius advancement flap for low rectovaginal fistula: short- and long-term results. Color Dis.

[28] Cilek NY, Mulayim BA (2012). Mullerian anomaly “without classification”: septate uterus with double cervix and longitudinal vaginal septum. Taiwan J Obstet Gynecol.

[29] Etzioni DA, Young-Fadok TM, Cima RR (2014). Patient survival after surgical treatment of rectal cancer: impact of surgeon and hospital characteristics. Cancer.

[30] Abcarian H (1999). The time has come. Tech Coloproctol.

[31] Tozer PJ, Balmforth D, Kayani B, Rahbour G, Hart AL, Phillips RK (2013). Surgical management of rectovaginal fistula in a tertiary referral centre: many techniques are needed. Color Dis.

